# Promoting emergent literacy in under-served preschools using environmental print

**DOI:** 10.4102/sajcd.v68i1.809

**Published:** 2021-05-17

**Authors:** Lauraine Giacovazzi, Sharon Moonsamy, Munyane Mophosho

**Affiliations:** 1 Department of Speech Pathology, Faculty of Humanities, University of the Witwatersrand, Johannesburg, South Africa

**Keywords:** collaboration, emergent literacy, environmental print, under-served preschools, SLT

## Abstract

**Background:**

Children from under-served communities are at risk for delayed spoken language and literacy development. Under-served preschools frequently contend with lack of resources, especially with regard to access to age-appropriate storybooks and/or print resources. Environmental print is a cost-effective material that can be used to stimulate emergent literacy skills. In the context of under-served communities, a collaborative approach and mentorship between preschool teachers and Speech-Language Therapists (SLTs) promote language and literacy development.

**Objectives:**

This article’s purpose is two-fold; firstly, to discuss the use of environmental print as a stimulus material to promote emergent literacy in preschoolers in under-served preschools. Secondly, to promote the SLT’s involvement in such education initiatives.

**Method:**

A mixed-method, comparative intervention research design, was reported in this article. A pre- and post-test design was employed, with data collected before and after a teacher-based intervention.

**Results:**

Participants in the intervention group displayed increased scores on the Concepts About Print (CAP) assessment, participants in the comparison group showed no change in scores using the same assessment over the same time period.

**Conclusion:**

A short-term, teacher-based intervention using environmental print with SLT mentoring and collaboration promoted preschool children’s emergent literacy skills. Implications include the value of using environmental print as a teaching material and the positive impact of collaboration between SLTs and teachers to promote emergent literacy in preschool children.

## Introduction

This article aims to highlight a section of the authors’ larger study to demonstrate the benefits of using environmental print as a cost-effective and successful stimulus material to teach print concepts and emergent literacy skills to children in under-served preschools. The article also promotes the collaboration of professionals such as Speech-Language Therapists (SLTs) with preschool teachers in under-served communities, further highlighting the need for explicit teaching practices that would benefit young children’s emergent literacy development. The concepts of environmental print and under-served preschools will be explored further in more detail.

Environmental print is the print that is found in public spaces (e.g. road signs, billboards), shopping areas (e.g. brand labels, restaurant signs, logos) and can extend into the school or work areas (e.g. posters) (Neuman & Celano, 2001; Neumann, Hood, & Ford, 2013; Vera, 2007). Environmental print can be a particularly valuable tool for use as a stimulus, encouraging the development of certain language skills and emergent literacy in young children. Environmental print is cost-effective, easily accessible and has frequent occurrences outside the classroom. Its availability outside the classroom allows parents to continue stimulating what their child had learned at school, consolidating their learning. Cost-effectiveness becomes a pivotal aspect when considering interventions to address teaching of emergent literacy skills in the South African context, as socioeconomic disparities are evident in our society.

Continuing inequalities in socioeconomic status, lack of access to a variety of resources for language and literacy learning in the classroom and even limited access to learning in one’s first language exist across South African communities (Graven, 2013). Such inequalities leave many children in South Africa at risk for spoken language and literacy developmental delays, which hinder their progress in education, placing them at risk (Fernald, Marchman, & Wiesleder, 2013; Hoff, 2006). Furthermore, the aftermath of apartheid and its effects that the majority of South Africans continue to face implies that socioeconomic status is multi-dimensional. Thus, describing the middle socioeconomic class can become complex, as it depends on the perspective and interpretation of the reader (Burger, Steenekamp, Zoch, & Van der Berg, 2014; Schotte, Zizzamia, & Leibbrandt, 2017; Schutte, 2018). Perspective taking, often makes it challenging to determine the potential influence of socioeconomic status on spoken language and literacy development in the South African context. Nonetheless, it is well-documented in research internationally that socioeconomic status influences children’s development (Fernald et al., 2013; Hoff, 2006). Therefore, persistent inequalities in the South African context will negatively impact language and literacy skills, unless explicit collaborative interventions are applied to break the cycle (Graven, 2013).

Children from lower socioeconomic and under-served communities are, therefore, frequently placed in a disadvantaged position, academically and socially when compared to the same aged peers from middle to upper socioeconomic communities (Walton, Bekker, & Thompson, 2015). The term *under-served* applies particularly to schools or communities that are not provided with the same facilities, resources or services that are typically found in middle to upper income communities and/or schools. Children in under-served preschools repeatedly contend with lack of resources in their classrooms, especially with regard to access to age-appropriate storybooks and/or print resources as well as access to support services from SLTs (Neuman & Celano, 2001). Limited access to resources is often largely related to cost. In the case of South Africa’s under-served preschools, they often cannot afford to purchase such resources, as they are not funded sufficiently by the government (relying on fees from parents to fund their schools). Hence, environmental print becomes a viable, cost-effective resource for teaching emergent literacy skills in these contexts.

There is a continuing and urgent need for supporting learners in language and emergent literacy development in the early years in South Africa (Graven, 2013). Methods of teaching that support language and literacy development in at-risk South African children require in-depth research from all stakeholders. Similarly, reasons for and ways to address language and literacy delays require more research in the South African context. Environmental print is a resource that could be used for teaching print concepts within the preschool setting with potentially positive outcomes for promoting children’s emergent literacy development. Collaborative support from SLTs for preschool teachers is an additional measure that would promote emergent literacy development in children, attending under-served preschools.

## Theoretical framework

For this study’s purpose, Vygtosky’s socio-cultural theory was selected as it underpins the language-learning approach adopted in the intervention employed. Socio-cultural theory focuses on three main areas: social interaction, the more knowledgeable partner (e.g. the adult in teacher-child interactions) and the zone of proximal development (ZPD) (Keenan & Evans, 2009). The emphasis Vygotsky placed on cultural and social interactions is apt for the hugely multi-cultural South African environment (Wieten & Hassim, 2016). Vygotsky’s theory advocates that the child plays an active role in his or her cognitive development together with the impact of social interactions with parents, teachers and older children who can provide invaluable guidance (Wieten & Hassim, 2016). The ZPD is defined as the distance between what children can do by themselves and the concepts and skills they can learn with the assistance from adults (Levey, 2014). Adult individuals aside from parents also include teachers, who play an important role in child development in the early years, within the school environment.

## Literature review

### Emergent literacy

Varied international and local literature supports the topics of language development, emergent literacy, literacy practices, parent- and teacher-based interventions and the effect of socioeconomic status on variation in these areas (Atmore, 2013; De Witt, 2009; Fernald et al., 2013; Heugh, 2009; Jordaan, 2011; Kathard et al., 2011; Wium & Louw, 2013). Literacy encompasses the skills of spelling, reading and writing (Wium, 2015) and can be viewed as a social practice influenced by the individual, home culture and school (McKenzie, 2015). Emergent literacy, a practice that begins before the formal instruction of literacy, involves both written language awareness and phonological awareness (recognising and working with the sounds used in spoken language), which are both based on typically developing oral language (Giess, 2010; Wium, 2015). Print concepts are part of emergent literacy development and according to Clay (2000), it includes if a child understands the following variables:

- Print has meaning.
- Print can be used for different purposes.
- There is a relationship between print and spoken language.
- There is a difference between letters and words.
- Words are separated by spaces.
- There is a difference between words and sentences.
- There are punctuation marks that signal the end of a sentence.
- Books have parts (front and back cover, title, page and spine).
- Stories have a beginning, middle and end.
- Text is read from left to right and top to bottom.

The print concepts listed above are included in Clay’s (2000) assessment tool, Concepts About Print (CAP). This particular assessment tool was used in this study at pre-testing and post-testing.

Beginning readers first develop early or emergent literacy skills and continue to develop these along a continuum until they have established these skills and become independent readers. Both literacy and language skills are dependent on a foundation of listening and language competence (Wium, 2015).

The main research indicates that the earlier literacy practices are introduced into children’s typical interactions with their parents or significant others, it is more likely that children will reap positive gains for their later literacy development (e.g. Dodici, Draper, & Peterson 2003; Griffin, Hemphill, Camp, & Wolf, 2004; Ntuli & Pretorius, 2005). Early exposure and or intervention hence become critical not only in home practices but also in educational settings like preschools. This would be especially crucial for at-risk children, which include children from under-served preschools.

Oral language skills play an essential role in literacy development as do concepts surrounding the print itself. Neumann (2016) found that Australian parents from more advantaged backgrounds seemed to teach their children more about print (teaching them specifically about letters and words) when compared to parents from lower socioeconomic backgrounds. Results like these further emphasise the potential differences that exist between children from varying socioeconomic backgrounds. Neumann’s (2016) results also suggested that a specific focus on explicit teaching of print concepts in home literacy practices should not be neglected. Print concept knowledge and explicitly teaching these concepts to young children aid in furthering emergent literacy both at home and at school.

Previous research in the field of emergent literacy has, therefore, proven that early exposure to literacy skills and explicit instruction are keys to building developmental foundations that promote long-term success in literacy progress (Griffin et al., 2004; Reutzel et al., 2003). For the purpose of this article, a focus on the South African context, given its history of inequalities, will give a clear view of the importance and need for support in language and literacy learning in the early years. Likewise, it is important to examine how SLTs can support learners and collaborate with teachers to further learner’s language and literacy development.

### The South African context and involvement of Speech-Language Therapists in education

There is an unrelenting need for interventions, aimed at promoting language and literacy development in preschool South African children (Kathard et al., 2011; Wium & Louw, 2013). In fact, an urgent need to address literacy across age ranges exists in South Africa (Gustafsson, Van der Berg, Sheperd, & Burger, 2009).

Many South Africans experienced disadvantages across contexts as the result of apartheid, including access to quality education, thus affecting literacy acquisition (Moonsamy, Mupawose, Seedat, & Mophosho, 2017). Apartheid was a policy of segregation based on race, violating the human rights and dignity of the majority, its black populations (Constitution of South Africa, 1996). The South African government has acknowledged that there are ‘unquestionable reasons’ for investing specifically in Early Childhood Development (ECD) (Republic of South Africa, 2010, p. 13). These reasons include children’s rights, human development, equity and the economy (Republic of South Africa, 2010). There has been an increase in children aged 0 to 4 years attending ECD centres (preschools) from 7% in 2002 to 30% in 2009 (Republic of South Africa, 2010). In 2016, 53% of children aged 0–6 years attended ECD centres across the country; however, disparities exist between urban and rural access (Statistics of South Africa, 2016). The government acknowledged that ‘much more is required to improve the quality of education provided’ (Republic of South Africa, 2010, p. 10). Literacy levels in particular go on to affect employment opportunities placing a person who may be illiterate or presenting with below average literacy skills at further economic disadvantage (Gustafsson et al., 2009). Literacy is, therefore, in a prominent position for evaluation and monitoring, locally and internationally.

Progress in International Reading Study (PIRLS) collects data to provide insight into reading achievements in the fourth, fifth and sixth grade across the globe (Mullis, Martin, Foy, & Hooper, 2017). South Africa’s performance in PIRLS assessments has placed the country’s literacy achievement below the international benchmark (Mullis, Martin, Foy, & Drucker, 2012; Mullis et al., 2017). These statistics imply that South Africa is not progressing in its reading achievement at the rate expected. Some of the factors identified as potentials for increased achievement in reading via the research gathered from PIRLS are summarised (Box 1).

BOX 1 Factors related to increased achievement in reading.

**Factors**

- Home environments that support literacy
- Attending well-resourced, academically orientated schools
- An early start to literacy instruction (either at home or by attending a pre-primary school)
- Increased teacher education and career satisfaction
- Attending school regularly without being hungry or tired
- Having a positive attitude towards reading

*Source*: Mullis, I.V.S., Martin, M.O., Foy, P., & Drucker, K.T. (2012). PIRLS 2011 international results in reading. Chestnut Hill, MA: TIMSS and PIRLS International Study Center; and, Mullis, I.V.S., Martin, M.O., Foy, P., & Hooper, M. (2017). *PIRLS 2016 International Results in Reading*. Boston College, TIMSS & PIRLS International Study Center. Retrieved from http://timssandpirls.bc.edu/pirls2016/international-results/

Box 1 presents factors that relate to reading achievement and are important to the South African context. Additionally, Mophosho, Khoza-Shangase and Sebole (2019, p. 62) listed the following as contributing factors to the development of reading: how much time is devoted to reading in schools, class organisation (numbers of learners and interactions between them and the teacher), methods used for reading as well as assessing progress, language spoken at home as well as exposure to the language of learning and teaching.

South African schools within middle to upper class communities tend to be resourced with materials, social resources, well-trained teachers and parents who are sufficiently affluent to support optimum conditions for language and literacy development. Such schools also have access to various therapists, including SLTs, occupational therapists, physiotherapists and psychologists. However, schools within under-served communities do not have these same advantages (e.g. townships, rural areas and some less wealthy urban areas) (Kathard et al., 2011; Walton et al., 2015).

Foundational to the concept of improving the quality of language and literacy teaching practices in schools is a focus on early interventions in ECD (preschools) rather than later in formal primary schooling. This early intervention is fundamental to young children’s developmental potential (Vally, 2012) during the critical development period (Owens, 2014). Further improving the quality of South African schooling requires increased access, better value education and an understanding of the multilingual context of South Africa as well as how children could benefit from it (Mophosho et al., 2019). The language of learning and teaching gives children access to navigating the curriculum effectively. In the context of preschool, this means access to developing essential language and emergent literacy skills, as prerequisites for later scholastic success. SLTs have the potential to contribute to this particular aspect of South African schooling, via teacher collaborations and mentoring (Moonsamy & Carolus, 2019).

SLTs are trained in understanding and providing interventions for language and literacy delays and disorders across age ranges. Understanding that the SLT has a vital role in collaboration with the education system is essential in providing relevant intervention and services within school contexts in South Africa (Kathard et al., 2011; Wium & Louw, 2013). Locally, there is a call for an increase in the involvement of SLTs in education to enhance language and literacy outcomes in South Africa (Moonsamy et al., 2017). Before the end of apartheid in 1994, SLTs were employed in schools for only white learners and in special schools for children with disabilities, regardless of race (Moonsamy et al., 2017). Post-1994, SLTs were not considered for inclusion in employment within the school system apart from special schools (constituting 3% of South African schools) (Moonsamy et al., 2017). This leaves the majority of learners, especially within the public school system without direct access to SLT services.

Notwithstanding the challenges of offering SLT services in schools, there is a call within the profession itself for SLTs to increase their involvement in the South African school system. Kathard et al. (2011) stated the following:

[T]he lens of the profession must expand from the traditional impairment-driven frame to a broader and more inclusive framework which considers not only those who have language and literacy learning impairments, but also considers those who are at risk for literacy development and hence educational failure as a result of disabling systemic conditions. (p. 61)

Wium and Louw (2013) expanded on the necessity for South African SLTs to take on a similar reform as was stated by the American Speech-Language Association (ASHA) in 2010 regarding four fundamental areas: critical roles, range of responsibilities, collaboration and leadership. SLTs have a key role to play in the education system and contribute to ‘education and preparing citizens of the future’ (Wium & Louw, 2013, p. 36). Children who face formal education without intact listening skills, spoken language skills and metalinguistic skills become at risk for academic failure. This, in turn, places them at risk for ‘low self-esteem, social maladjustment and ultimately vocational problems’ (Kathard et al., 2011; Wium & Louw, 2013, p. 33). However, SLT’s contributions towards supporting learners do not have to be limited to directly working with learners but could contribute to supporting teachers. SLTs have the opportunity to not only work with teachers in terms of language-literacy teaching and learning but also to promote resourcefulness in selecting cost-effective materials, like environmental print.

### Environmental print

Environmental print is a valuable resource that can be used in a learning environment where children may be learning in their second language. This has been demonstrated in both Vera (2007) as well as Bhuvaneswari and Pradakannaya (2017) studies with learners speaking English as their second language but attending English medium schools. With South Africa’s multilingual nature, tools that are flexible for learning in multiple languages are prized. In education, one important component contributing to children’s literacy development is a classroom environment rich in written language.

There have been limited published studies in South Africa which investigate the possible changes an environmental print intervention could make in children’s literacy skills from under-served communities. This study, therefore, aimed to address this knowledge gap. Specifically, Neumann et al. (2013) and Vera’s (2007) studies were explored as an adapted model of intervention for the current study. Both of these studies prove valuable in confirming the potential of environmental print in teaching print concepts as a part of emergent literacy development. Environmental print has several advantages including cost-effectiveness, accessibility and has frequent occurrence outside the classroom. Neumann et al. (2013) findings suggested that environmental print might be more effective than standard print when teaching print concepts, enhancing print motivation and increasing some aspects of emergent literacy because of its contextual nature. Participants who were learning English as the second language in Vera’s (2007) study furthermore expanded their alphabet knowledge in the context of English and this promotes the value of using environmental print in multilingual contexts (such as South African preschools). The current study aimed to improve emergent literacy skills of South African preschoolers through an environmental print intervention.

## Research methodology

### Research question

What is the effect of a teacher-based, SLT-supported, environmental print intervention on the emergent literacy skills of children in an under-served preschool?

The study investigated if any changes occurred in preschool children’s spoken language and emergent literacy skills, following a short-term teacher-based intervention using environmental print. This article focusses specifically on changes shown by individual children in the intervention group in terms of their emergent literacy print concepts at pre-testing and post-testing.

### Research design

A mixed methods comparative intervention research design was utilised, with a pre- and post-test design applied to measure print concepts. The CAP assessment was used pre and post the intervention phase. The researcher, (an SLT) and the ECD teacher dynamically created the intervention content weekly. In the Results section, the intervention is described (Table 4). Using a mixed methods approach allows the researcher to use multiple tools to gather more comprehensive data that cover a topic broadly (Creswell, 2009; Hunter & Brewer, 2015). Quantitative and qualitative data were collected. An overview of the data collection tools and data analysis is given (Table 1).

**TABLE 1:** Research methodology.

Variable	Tests	Assessments	Interviews	Observations
Tools	Pre- and Post-test measures: Concepts About Print (Marie Clay, 2000) Mean Length of Utterance and Type Token Ratios	Pre- and Post-test measures: Age-appropriate storybook	Self-developed questionnaires for teachers	Fidelity criteria checklist
Data Sets	Raw scores	Spontaneous narrative language sample	Open- and closed-ended questions	Criteria rating
Nature of Data	Quantitative	Quantitative and qualitative	Qualitative	Quantitative
Data Analysis	Descriptive and inferential statistics	Descriptive statistics, narrative analysis	Thematic content analysis	Descriptive and inferential statistics

Whilst an overview of the tools, assessments, interviews and observation methods used for the study as a whole is given (Table 1), this article only focuses on print concepts as it relates to emergent literacy. The CAP assessment tool is explained further for this purpose.

### Measures: Concepts about print

CAP assessment tool is based on Marie Clay’s (2000) research, as a means to ‘observe what children notice about written language in their environments’ (as cited in Johnson, 2015, p. 54). The CAP assessment is used to assess various print concepts (listed on p. 6.) This tool is efficient in its administration and has been researched and utilised in several different studies (including Johnson, 2015; Neumann et al., 2013; Tafa, 2009; Vera, 2007). Tafa (2009) found that the CAP assessment was highly reliable when used in Greek, demonstrating the adaptability of this tool into a different culture and language. This suggests a potential value for the South African context and proved effective within the current study. CAP comprises a checklist-style scoring sheet. Raw scores were tallied with the use of the assessment checklist. The checklist was used with an age- and culturally appropriate storybook. Children were probed with questions or instructions regarding specific concepts surrounding print. Two different storybooks were used: one at pre-test and one at post-test data collection. The books used were: ‘Wake Up, Mummy!’ by Stephanie Moss and ‘Who’s in My Family? All About Our Families’ by Robie H. Harris. The books were selected based on how the print would relate to the CAP assessment tool, together with the needs of the narrative sample (mentioned in Table 1). The CAP assessment has been found to be flexible as a tool used in various contexts, cultures and languages. The context of the current study’s participants is detailed below.

### Participants’ context

**FIGURE 1:**
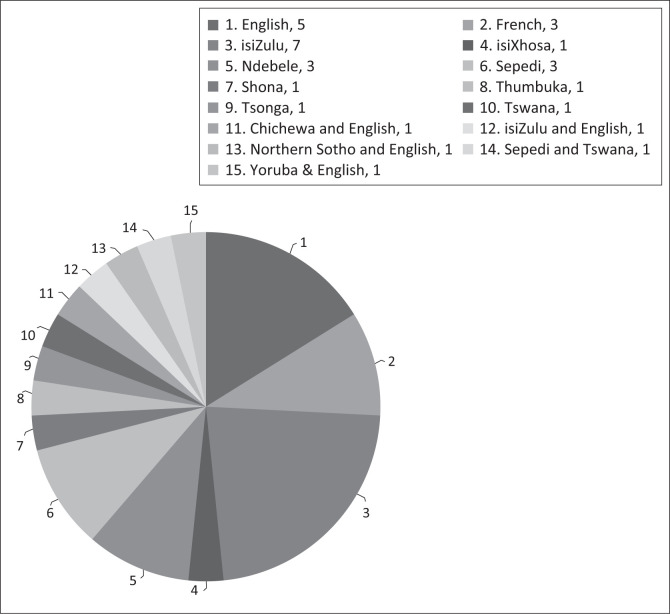
Reported first languages of learners.

A purposive sampling strategy was used to strategically create a sample that would be relevant to the research questions (Creswell, 2009). The Department of Social Development (DSD) assisted the researcher in sourcing two schools appropriate for the study. Both schools were located in the same area near the Randburg central business district (CBD), Johannesburg, South Africa. The schools were randomly assigned as either the intervention group or the comparison group before meeting with the teachers and principals in order to reduce potential researcher bias. All included participants gave informed consent in writing. Two teachers agreed to participate in the study. Both the intervention group and comparison group teachers had a Further Education and Training Certificate: ECD (NQF Level 4) qualification. The teachers’ first language was isiZulu, but the medium of instruction in both classes was English (their second language). Both groups of children spoke a variety of different languages as their first languages, including languages from other African countries (e.g. French, Chichewa, Shona, Yoruba, Thumbuka – refer to Figure 1). Further background information of the 32 children and their parents across both groups was gathered via the consent forms. Information specific to the children’s developmental profiles is demonstrated (Table 2) and (Figure 1), as the contextual information was important when creating the intervention sessions.

**TABLE 2:** Children’s reported background information.

Variable	Yes	No
At least one employed parent	28	3
Children with known cognitive impairments	0	32
Children with known visual impairments	0	32
Children with known hearing impairments	0	32
Children attending Speech-Language Therapy	1	31

### Data collection

**FIGURE 2:**
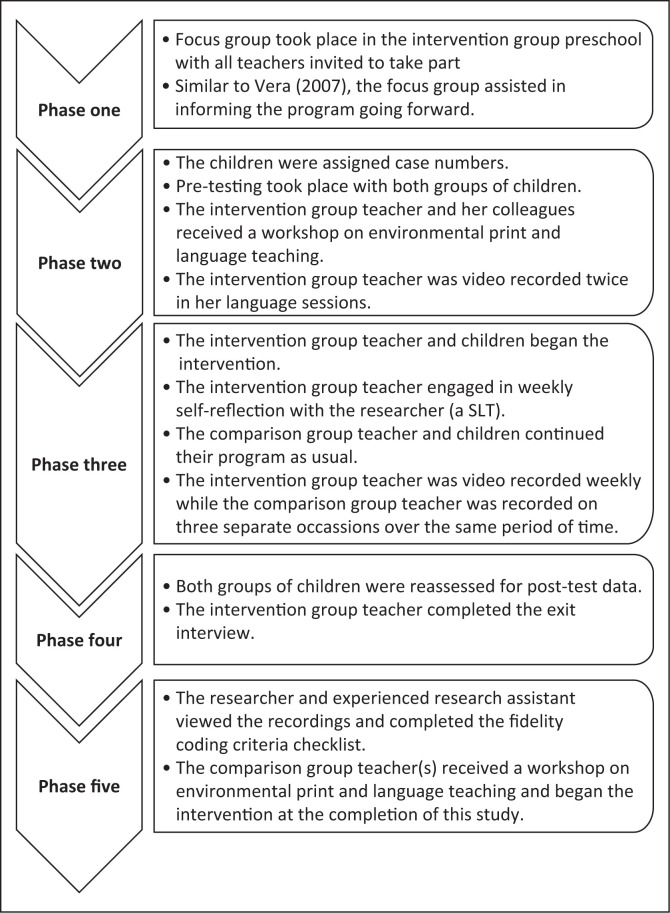
Data collection procedures.

Data collection took place across five phases as described (Figure 2).

**FIGURE 3:**
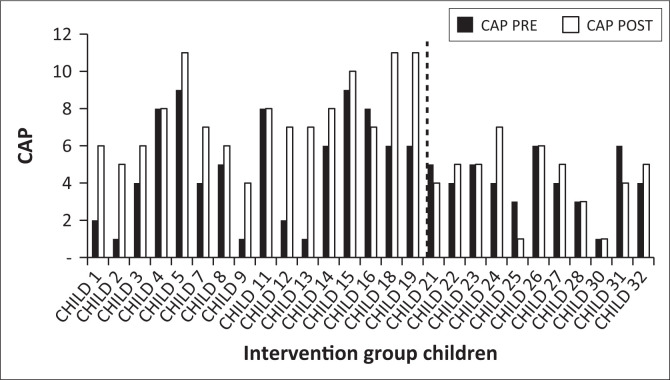
Concepts About Print (CAP) pre- and post-test results (both groups).

Pre-test and post-test CAP assessment scores were collected in phases two and four, respectively, as shown (Figure 2). The data were analysed using descriptive statistics (Figure 3) and inferential statistics (Table 4).

### Ethical considerations

This research was cleared by the Human Research Ethics Committee (Non-Medical) at the University of the Witwatersrand, reference number: H17/07/06.

## Results

For the purpose of this article, results presented relate specifically to the intervention sessions and the pre- and post-test data on the CAP assessment. Results from pre- and post-testing demonstrated statistically significant increases in scores on the CAP assessment in the intervention group compared with the comparison group. The comparison group did not display statistically significant increases in CAP scores over the same period of time.

An outline of the 12 intervention sessions in terms of activities and materials used is provided (Table 3).

**TABLE 3:** Intervention sessions.

Session Number	Activities	Materials
1	Explicit instruction within a ‘weather’ theme† Drawing activity	Classroom posters Handmade name cards‡
2	Explicit instruction within a ‘weather’ theme† Drawing activity	Classroom posters Magazine and newspaper adverts Classroom blackboard Handmade name cards‡
3	Explicit instruction within a ‘shopping’ theme† Kinetic activity where children were required to locate their name cards Presenting collected materials	Recycled, empty grocery boxes, cartons, tins, bags, bottles (collected by the children, teachers and researchers) Handmade name cards‡
4	Explicit instruction within a ‘shopping’ theme† Actively arranging items into categories in teams	Recycled, empty grocery boxes, cartons, tins, bags, bottles (collected by the children, teachers and researchers) Handmade name cards‡
5	Explicit instruction within a ‘shopping’ theme† Art activity creating ‘money’ for the children’s pretend grocery store	Example coins and notes with pictures gathered from available magazines or newspapers‡ Scrap paper, scissors, crayons
6	Play activity with a pretend grocery store set up in the classroom Assigned role play that each child had the opportunity to explore (e.g. shoppers, cashiers, staff)	Recycled grocery items collected over lessons 3 and 4, pretend money from lesson 5, paper wallets with names and shopping lists (made with the teacher between lessons 5 and 6)
7	Explicit instruction within a ‘road signs, transport and traveling’ theme† Art activity creating road signs (stop signs, street signs and building signs (e.g. ‘school’, ‘park’, zoo), stop and pedestrian crossing signs, traffic robots	Recycled grocery items collected over lessons 3 and 4 (which were cut and painted over) Paint and paintbrushes
8	Play activity on the school playground using the created road signs and traffic robots Assigned role play that each child had the opportunity to explore (e.g. drivers, pedestrians, policemen)	Created signs from lesson 7
9	Explicit instruction within a ‘newspapers’ theme† Small groups of children explored newspapers for letter-sound associations, locating parts of the text related to titles (bigger text) versus stories (smaller text) and pictures versus text.	Recycled free local newspapers collected from the school and children
10	Explicit instruction within a ‘newspapers’ theme† Small groups of children used the newspapers explored in lesson 9 to recreate their own ‘newspaper’ and create the story within it.	Recycled free local newspapers collected from the school and children Scrap paper, glue, scissors, crayons
11	Explicit instruction focussed on listening skills and phonemic awareness† Kinetic activity where children were given instructions to follow a path (e.g. ‘jump’ on the square, circle and triangle). The children then were required to choose a letter, the phoneme associated to it was reinforced, the children matched the phoneme to a picture with same phoneme in the initial position of the word (letters vs. pictures)	Letters ‘a/A’, ‘m/M’, ‘s/S’, ‘t/T’ and ‘p/P’ and corresponding pictures were cut out from the leftover items‡ collected over the course of the intervention Soft plastic shapes (belonging to the preschool)
12	The intervention teacher was not satisfied with session 11 and opted to repeat the session plan in session 12 with some adjustments: The children sat at tables The teacher used the pictures and letters as an explicit demonstration The children chose a letter/phoneme and drew matching pictures of words they generated.	Letters ‘a/A’, ‘m/M’, ‘s/S’, ‘t/T’ and ‘p/P’ and corresponding pictures were cut out from the leftover items‡ collected over the course of the intervention Scrap paper, crayons

Notes: For photographic examples see (Figure 4).

† , Explicit instruction involved the teacher explaining and detailing concepts to the children before they engaged in the designated activities.

‡ , The intervention teacher prepared this item before the session.

The intervention sessions planned weekly, with the teacher were presented (Table 3). Each session utilised environmental print materials as well as explicit instruction of the concepts taught in each session. The pre- and post-testing scores for CAP across both groups of children (with children 1 to 19 representing the intervention group and children 21 to 32 representing the comparison group) are displayed (Figure 3).

Gains at post-testing appeared more frequently amongst the intervention group when compared with the comparison group children as shown (Figure 3). This was confirmed via the paired sample *t*-tests; the *p* values for the null hypotheses are demonstrated (Table 4).

**TABLE 4:** Concepts About Print *t*-test *p* values (Intervention and Comparison Groups).

Intervention group	Comparison group
*P* (*T* ≤ *t*) one-tail: 0.000077	*P* (*T* ≤ *t*) one-tail: 0.419506578
Null hypothesis was rejected at 1% level of significance	Null hypothesis could not be rejected, even at 10% level of significance

For the intervention group children, the null hypothesis was that the mean CAP scores at pre- and post-testing were equal. The alternative hypothesis was that the mean CAP score at post-testing was higher than the mean CAP score at pre-testing (one-tailed test). Using the data, the null hypothesis was rejected at even a 1% level of significance (0.000077 < 0.01) as shown (Table 4). From the data, it can be concluded that the mean CAP score at post-testing was greater than the mean CAP score at pre-testing amongst the intervention group children. A paired two-sample *t*-test was used to test whether the mean CAP score at post-testing is higher than the mean CAP score at pre-testing amongst the comparison group children. The null hypothesis was that the mean CAP scores at pre- and post-testing were equal. The alternative hypothesis was that the mean CAP score at post-testing was higher than the mean CAP score at pre-testing (one-tailed test). Using the data, the null hypothesis could not be rejected at even a 10% level of significance (0.4195 > 0.1) as shown (Table 4). There was, therefore, insufficient evidence to suggest that the mean CAP score at post-testing was greater than the mean CAP score at pre-testing amongst the comparison group children.

The comparison group children did not make similar gains in CAP scores within the same time frame as the intervention group children. Again, one can deduce that the increases that the intervention group children presented with on the CAP assessment at post-testing may be attributed to the intervention. Both the classrooms of the intervention and comparison groups displayed some environmental print in the form of posters, often hand-made ones. According to Reutzel et al. (2003), it is not enough to simply display environmental print in the classroom; rather one needs to explicitly teach children to apply word study, decoding skills and phoneme-grapheme knowledge using the environmental print as a material. Explicit instruction using environmental print was included in the intervention (Table 3). This particular interpretation, along with other salient topics is included in the discussion section.

## Discussion

Using environmental print was an effective means to sourcing cost-effective materials to teach pivotal print concepts (refer to Table 3 for intervention details). The environmental print that was used within the intervention comprised of materials that the teachers and learners collected or was readily available in the classroom (e.g. posters). The teacher and the researcher were able to maintain a cost-effective approach by becoming resourceful with what was available to the intervention at no financial cost (Vally, 2012). Environmental print is cost-effective because one can source materials by recycling packaging goods (e.g. cans, cereal boxes, bottles amongst others) or view it at no cost in the form of public signs. Environmental print allows children to learn the variables of print concepts (listed on p. 2) with a similar efficacy to traditional print resources (e.g. storybooks) (Neuman et al., 2013).

**FIGURE 4:**
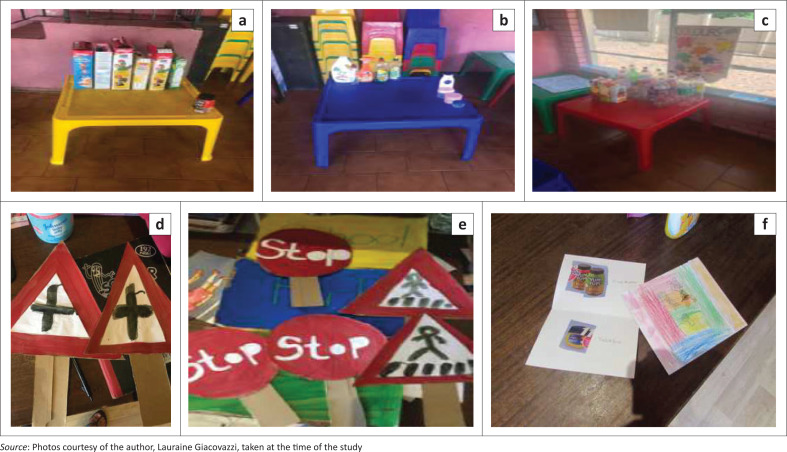
Photos of environmental print materials and recycled (‘new’) materials: (a), (b), and (c) are examples of the materials collected; (d), (e) and (f) are examples of how the materials were reused.

It is already known that children in under-served preschools often face lack of resources in their classrooms (e.g. age-appropriate storybooks and/or print resources) (Neuman & Celano, 2001). Both schools had few print resources in the form of printed and hand-made posters; however, the teachers were not maximising the print materials’ inclusion at pre-intervention. The researcher through collaboration had the opportunity to foster resourcefulness in collecting the environmental print material for teaching language and emergent literacy skills. Teachers and learners collected materials by recycling empty packages, boxes, cans or similar materials from their homes and schools (refer to Table 3 and Figure 4 for examples).

Using environmental print created a viable opportunity to use print that the children would likely encounter again outside of the intervention, lending to the high impact on recognition that environmental print offers (Neumann et al., 2013; Vera, 2007). Carryover of some of the lesson content to the home environment was observed when parents began reporting to the intervention teacher that their children were asking questions, drawing attention to and making statements about print they saw at home and in shopping areas particularly (e.g. brand labels, shopping lists, print found on bank notes or coins).

Consequently, the intervention teacher was presented with the opportunity to talk to parents about what materials she was using in the classroom, as the children had initiated conversations and interactions outside the classroom with their parents about their lesson content. Teacher and parent partnership holds an important influence over a child’s learning as well as the teacher and school as a whole (Henderson & Berla, 1994). Gonzalez-DeHass, Willems and Doan Holbeim (2005) noted that significant interactions between parents and teachers positively influence a child’s efforts, concentration and school attendance. Hence, the increased interaction of the intervention teacher with parents should be seen as a positive gain. It is important to reiterate that it was not merely the presence of the environmental print that made the children’s gains possible but the teacher’s purposeful and explicit use of the material and teaching technique.

Instruction, therefore, needs to be explicit and intentional. The intervention group children experienced an intervention where their teacher used more explicit instruction on skills relating to concepts about how print works and phonological awareness skills as opposed to the comparison group children within this particular time frame (see Table 4). Moonsamy and Carolus (2019) found, similarly, an increase in CAP scores at post-testing following a literacy intervention programme with South African ECD or preschool teachers. In this particular study, the researcher collaborated with teachers to promote explicit instruction on print concepts through varied reading strategies. Hence, Moonsamy and Carolus (2019) further promoted not only the need for explicit instruction but also the benefits of SLTs and teacher collaboration. Moonsamy and Carolus (2019) used storybooks as the resource for explicit instruction whilst Neumann et al. (2013) and Vera (2007) found that CAP scores amongst their participants also increased at post-testing following environmental print interventions, specifically. These examples of explicit instruction and purposeful interaction with environmental print materials support the argument of the current study that collaborating with the teacher on environmental print creates opportunities for children to promote their emergent literacy skills.

Environmental print materials as an educational tool have shown the possibilities of a resource that is cost-effective, flexible in its use for multiple activities and relevant to the group of children who collect the materials from their own direct environments. Increases at post-testing in CAP scores amongst the intervention children were statistically significant and likely attributed to the intervention as a similar trend was not observed amongst the comparison group children. These improvements demonstrated that the children were able to generalise the print concepts they had acquired via the environmental print materials to book materials (used with the CAP pre-test and post-test). A similar finding was also presented by Neumann et al. (2013) and Vera (2007). This finding reinforces the theory of the interaction within the ZPD between the teachers and the children in their classroom whilst learning print concepts. The findings also supported the theory that environmental print can be used as an effective resource for teaching preschool children print concepts successfully.

## Conclusion

The study has revealed that collaboration between SLTs and teachers can make a positive impact on promoting language and emergent literacy skills in preschool children. The SLT’s specific knowledge and skill combined with mentoring and collaborating with teachers can contribute towards the current urgent needs surrounding maximising cognitive potential in South African preschoolers. Short-term interventions continue to show their value in stimulating literacy and language skills, as well as, promoting SLTs involvement in education. Short-term interventions create multiple opportunities for SLTs to make a positive impact with regard to promoting language and emergent literacy teaching and potentially to increase the skills in preschoolers. Environmental print offered itself as a valuable resource for teaching language and emergent literacy skills amongst the children who participated in the study. The implication of using environmental print as a teaching material should motivate professionals working in education (especially in the preschool context) to be resourceful in the face of limited traditional resources. The high impact that environmental print offers within the classroom cannot be under-estimated.

The principles of language and emergent literacy teaching continue to be at the forefront of discussion, as we address the need to maximise South African children’s cognitive potential (especially within under-served communities). The theoretical principles of the ZPD were demonstrated within the current study between the teachers and their preschool learners. Furthermore, the theory that environmental print is effective in teaching print concepts to preschool children was reinforced. This particular finding is pivotal for under-served populations that lack resources to teach print concepts (such as story books or other print resources that may be costly).

## Strengths of the study

The statistically significant results of the CAP that triangulated with qualitative data were a valuable strength to this study. Having a student research assistant assist in collecting pre- and post-test data also offset potential researcher bias. In addition, the researcher (and assistants) had no previous relationship to the schools, which reduced further bias. The DSD assisting with the selection also helped to match the schools, teachers and learners as closely as possible. The researcher working closely with the DSD supports the concept of collaboration between government departments, schools and researchers, so that evidence-based practice can emerge. Furthermore, the two schools participating within the study meant that the researcher could use an intervention and comparison group design. Comparing results in this manner added validity to outcomes, as the researcher was able to infer that changes observed were likely linked to the intervention.

## Limitations of the study

Whilst every effort was made to strengthen the research design, limitations did exist and should be acknowledged when considering the study’s results and interpretation. Having a smaller sample size means that results cannot necessarily be generalised to all groups of children; however, the information gained contributes to the clinical and theoretical implications. The researcher and the student research assistant were not fluent in other South African languages and this could also be seen as a limitation. However, there were multiple languages that were spoken by the children, attending this school, as they hailed from various countries in Africa.

Even though the results of the current study are not generalisable to other contexts, the findings have powerful contributions to the value of environmental print as a teaching material. Furthermore, the outcomes of the study advocate mentorship and collaboration between SLTs and teachers in ECD centres to promote preschool children’s language and emergent literacy skills. Furthermore, it contributes to advancing early intervention practices and promoting the SLT’s role in education.

## Recommendations for future research

Further research in this field could benefit the professions of both SLTs and preschool educators. Projects using environmental print, mentorship and collaboration performed with larger groups of participants could assist with the validity of the results. Application of multiple South African languages in short- or long-term environmental print interventions would also be valuable. In this manner, one would be able to ascertain the gains that could be made in children and teachers when they are placed in a position of advantage with their first languages.
